# Specialized bark-gnawing beetles reveal phragmotic defence and subcortical ecology in the Cretaceous

**DOI:** 10.1098/rspb.2025.1004

**Published:** 2025-06-11

**Authors:** Yan-Da Li, Richard A. B. Leschen, Jiří Kolibáč, Michael S. Engel, Zhi-Qiang Zhang, Yali Yu, Diying Huang, Chenyang Cai

**Affiliations:** ^1^ State Key Laboratory of Palaeobiology and Stratigraphy, Nanjing Institute of Geology and Palaeontology, Chinese Academy of Sciences, Nanjing 210008, People's Republic of China; ^2^ Bristol Palaeobiology Group, School of Earth Sciences, University of Bristol, Bristol BS8 1TQ, UK; ^3^ New Zealand Arthropod Collection, Manaaki Whenua Landcare Research, Auckland 1072, New Zealand; ^4^ Department of Entomology, Moravian Museum, 627 00 Brno, Czech Republic; ^5^ Division of Invertebrate Zoology, American Museum of Natural History, New York, NY 10024, USA; ^6^ School of Biological Sciences, University of Auckland, Auckland 1072, New Zealand; ^7^ Guangdong Key Laboratory of Animal Conservation and Resource Utilization, Guangdong Public Laboratory of Wild Animal Conservation and Utilization, Institute of Zoology, Guangdong Academy of Sciences, Guangzhou, People's Republic of China

**Keywords:** Cretaceous, beetle, phragmosis, defence, ecological interaction

## Abstract

Ecological interactions are fundamental to understanding species’ trophic relationships and the evolution of ecosystem functions. However, the fossil record seldom captures these intricate dynamics, as most fossils preserve individual organisms rather than the interactions that shaped ancient ecosystems. Here, we describe a new genus of bark-gnawing beetles (Trogossitidae), *Rutrizoma* gen. nov., from mid-Cretaceous amber in northern Myanmar. This fossil genus reveals a rare combination of predatory and antipredatory adaptations, shedding light on the ecological complexity of Mesozoic forest ecosystems. *Rutrizoma* has specialized morphological features, such as shortened elytra and unidentate mandibles, suggesting an active predatory lifestyle in narrow wood galleries. Interestingly, some morphological traits of *Rutrizoma* mirror those of its potential prey, particularly bostrichid beetles, from the same amber deposit. One such trait is its specialized abdominal declivity, which probably functioned as a protective shield against predators and competitors, representing marked convergence with the elytral declivity of other subcortical beetles, such as bark and ambrosia beetles (Scolytinae and Platypodinae) and Bostrichidae. The presence of phoretic mites associated with *Rutrizoma*, along with co-preserved bostrichid prey, underscores the complex community dynamics beneath Cretaceous tree bark. This finding reveals a subcortical ecosystem that parallels modern ecological interactions.

## 1. Introduction

One basic fact of life is to eat or be eaten. Indeed, predation is one of the fundamental interactions within ecosystems, driving the evolution of both predators and prey. Predators have developed a wide array of physical adaptations for detecting and capturing prey, while prey species have evolved corresponding defence mechanisms to avoid being caught [1]. Evidence of these predatory and defensive strategies is well-documented in the insect fossil record [2–5].

In recent years, numerous exciting discoveries have unveiled compelling evidence of predatory interactions preserved in mid-Cretaceous Kachin amber. Among these, the rove beetles *Cretobythus* Yin *et al.* and *Festenus* Żyła *et al.*, along with the larvae of the ground beetle *Loricera* Latreille, possess specialized sticky appendages (enlarged maxillary palpi, elongate protrusible labium and elongate galeae, respectively) to capture and deliver prey within striking range of the mandibles [6–9]. The adults of *Loricera* and the rove beetle *Clidicostigus* Jałoszyński *et al.* similarly possess stout setae on their antennae, an adaptation thought to assist in trapping prey [8–10]. Specialized raptorial legs have been found in various insect groups, particularly in the mantises [11,12] and raptorial lacewings [13–15].

To counter predation, many insect lineages evolved independently remarkable camouflage and mimicry strategies to avoid detection. The lacewing larva *Phyllochrysa* Liu *et al.*, the orthopteran *Phyllotridactylus* Xu *et al.* and the stick insect *Elasmophasma* Chen *et al.* all possess inflated or explanate structures mimicking plant elements [16–19]. Nymphs of certain hemipteran bugs, bark lice and various lacewing larvae carry debris as a form of disguise, effectively cloaking themselves from predators [5,18,20–23]. The flattened body with undulating tegminal margins and veins seen in some planthoppers further exemplifies this camouflaging capability [24]. Insect–insect mimicry has also been documented in alienopterid mantodeans, hemipteran bugs and true flies, with at least some of these instances likely serving defensive purposes [25–27]. The wing eyespots found in kalligrammatid lacewings may have intimidated potential predators [28]. Chemical defences are suspected in soldier beetles like *Ornatomalthinus* Poinar & Fanti, which may have been capable of excreting protective substances [29,30]. A possible anti-predator role has also been proposed for bioluminescence in lampyroid beetles such as *Cretophengodes* Li *et al.* [31,32]. These examples from Cretaceous amber provide a rare glimpse into the complex predator–prey dynamics of ancient ecosystems, highlighting the complex evolutionary arms race between predators and their prey in the late Mesozoic.

Within an ecosystem, most predators are simultaneously prey for others, leading progressively to an apex predator for a given community. However, most of the examples discussed above focus on only one side of these complex interactions. A notable exception is lacewing larvae, which possess conspicuous stylets for predation but are also masters of camouflage [5,20–22]. This dual adaptation allows them to hunt effectively while avoiding becoming prey themselves. Here we describe a new genus of bark-gnawing beetles, which not only possesses the capability to hunt within narrow galleries beneath tree bark but has also evolved a shield-like declivity at the posterior end of its abdomen for protection against predators or competitors. Remarkably, this defensive adaptation closely mirrors the specialized declivity seen in its own prey, highlighting the intricate ecological interactions between predator and prey in the Cretaceous subcortical ecosystem.

## 2. Systematic palaeontology

Order Coleoptera Linnaeus, 1758

Family Trogossitidae Latreille, 1802

Subfamily Trogossitinae Latreille, 1802

Genus ***Rutrizoma*** Li & Cai gen. nov.

**Type species**. *Rutrizoma donoghuei* sp. nov., here designated.

**Etymology**. The generic name is formed based on the Latin *rutrum*, shovel, referring to the bordered declivity at the abdominal apex, and part of the name *Nemozoma* Latreille, a closely related trogossitid genus. When Latreille established the genus name *Nemozoma* [33], he likely intended it to reference the elongate body (*νῆμα* [*nema*]: thread + *σῶμα* [*soma*]: body). Although Latreille improperly latinized the second component, since *ζῶμα* (*zoma*) is still a Greek neuter noun, according to ICZN Article 30.1.2 [34], *Nemozoma* should be treated as neuter (S. Laplante & P. Bouchard 2025, personal communication; contrary to [35]). The new name *Rutrizoma* should thus also be neuter.

**Diagnosis**. Body slender. Frons with weak longitudinal median groove, without paired horn-like processes. Antennae with 11 antennomeres; club weakly asymmetrical; antennomeres 9 and 10 without exposed sensorial field; antennomere 11 with exposed sensorial field. Mandibles with one prominent apical tooth. Galea with ciliate setae. Pronotal disc elongate, with longitudinal median groove. Procoxal cavities narrowly separated, closed externally. Elytra strongly shortened, apically truncate, leaving abdominal segments IV–VII fully exposed, each with five distinct longitudinal grooves. Mesocoxal cavities narrowly separated, open laterally. Metacoxae contiguous, medially projecting, laterally not reaching lateral margin of metathorax. Protibia with robust spine on mesal edge. Abdominal tergite VII specialized, forming bordered declivity; margins with dense ciliate setae. Abdominal ventrites strongly convex.

**Description**. Body slender; surface distinctly punctate, with metallic blue colour (best shown in fig. 1i in [36]).

**Figure 1.**
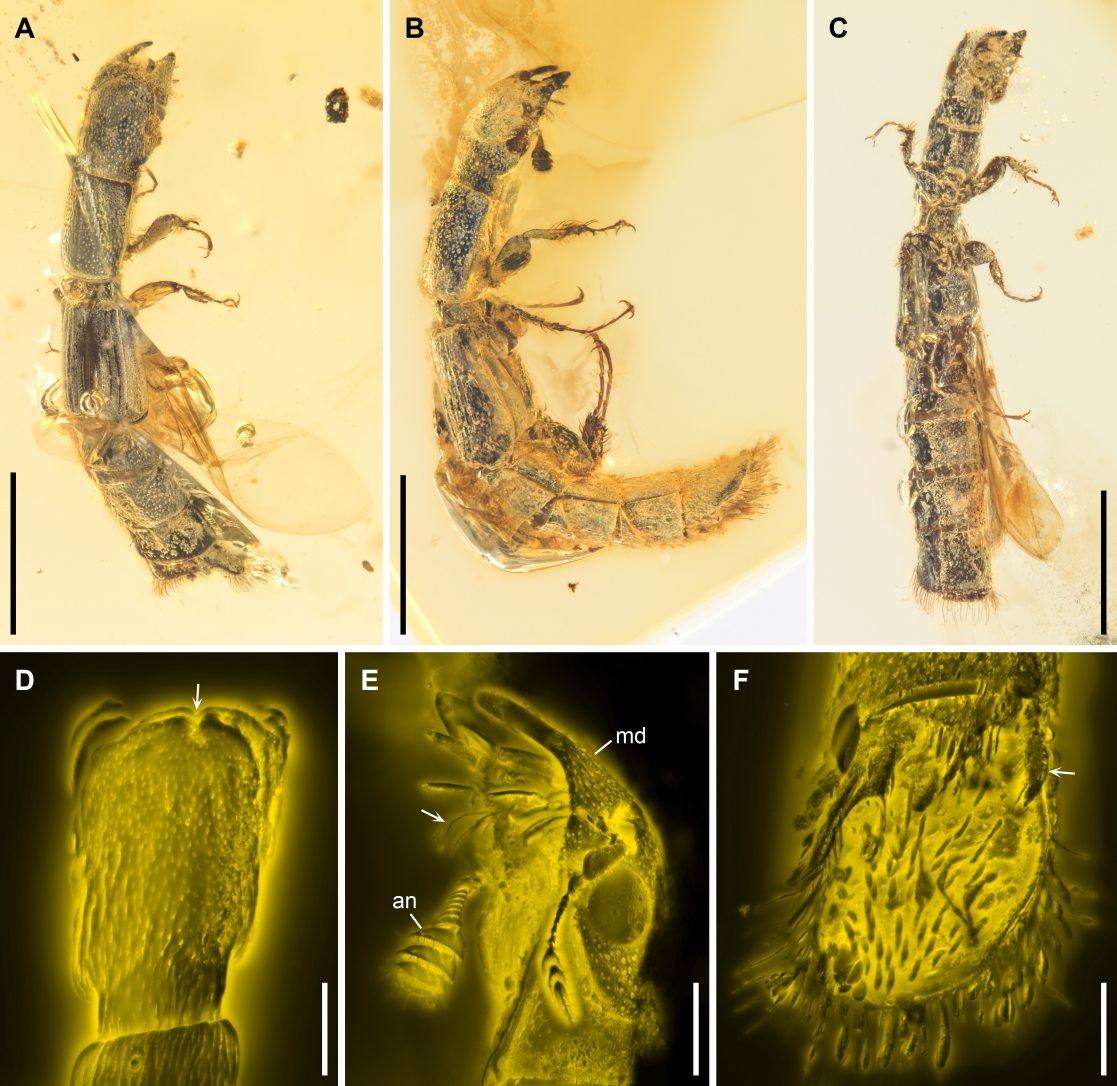
*Rutrizoma donoghuei* sp. nov., under brightfield (A–C) or confocal (D–F) microscopy. (A) NIGP205681, dorsal view. (B) NIGP166142, lateral view. (C) NIGP166143, ventral view. (D) NIGP205682-1, head, dorsal view, with arrow indicating median groove on frons. (E) NIGP166142, head, ventrolateral view, with arrow indicating ciliate setae of galea. (F) NIGP166142, abdominal declivity, dorsal view, with arrow indicating projected lobes. Abbreviations: an, antenna; md, mandible. Scale bars: 1 mm in (A–C); 200 μm in (D–F)*.*

Head prognathous, elongate, slightly wider than pronotum. Frons with weak longitudinal median groove, without paired horn-like processes. Frontoclypeal suture absent. Compound eyes moderate, not emarginate, situated laterally, relatively flat, not exceeding contour of cranium. Antennal grooves present in front of compound eyes. Antennae with 11 antennomeres; antennomere 1 robust, antennomere 2 smaller than 1 but larger than 3; antennomere 3−8 submoniliform, progressively slightly widened; antennomeres 9−11 enlarged, flattened, weakly asymmetrical, forming distinct club; antennomeres 9 and 10 without exposed sensorial field, apically bordered with dense setae; antennomere 11 apically with sensorial field. Mandibles large, with one prominent apical tooth and one medial tooth. Galea with ciliate setae. Apical maxillary palpomere elongate, cylindrical to subconical. Ventral surface of head without any tufts of setae. Gular sutures not visible.

Pronotal disc elongate, subparallel laterally; anterior edge nearly straight in dorsal view, finely crenulate; anterior and posterior corners nearly right-angled in lateral view; lateral pronotal carinae reduced, indistinct. Notosternal suture well-marked along full length. Prosternum in front of coxae relatively long; prosternal process complete, not dilated at apex. Procoxal cavities very narrowly separated, closed externally. Protrochantins hidden or absent. Mesonotum on peduncle distinctly punctate; mesoscutellar shield small, impunctate. Elytra strongly shortened, truncate apically, leaving abdominal segments IV–VII fully exposed, each with five distinct longitudinal grooves; groove 2 (counted from elytral suture) present only in posterior half; other grooves (almost) complete along full elytral length; groove 1 turning laterally at posterior end and continuous as transverse groove along posterior elytral edge. Hind wings well developed. Mesocoxal cavities very narrowly separated, open laterally (bordered partly by mesepimeron). Metaventrite broad; discrimen absent or very weak; katepisternal (paracoxal) suture absent; postcoxal lines absent. Exposed portion of metanepisternum elongate. Metacoxae contiguous, somewhat projecting medioposteriorly, laterally not reaching lateral margin of metathorax. Legs slender, with ciliate setae on tibiae and tarsi. Tibiae with large, robust spines along side; hooked spur present. Tarsi 5-5-5, elongate; tarsomeres simple; tarsomeres 2, 3 and 5 longer than tarsomeres 1 and 4. Pretarsal claws thickened at base; empodium projecting, bisetose.

Abdomen with tergites IV–VII visible dorsally (tergite III scarcely visible) and sternites III–VII (ventrites 1−5) visible ventrally. Tergites IV–VI with longitudinal medial depression and oblique grooves extending to anterior corners of tergite. Tergite VII concave, forming bordered declivity; margins with dense ciliate setae; surface sometimes with setae visible. Ventrites strongly convex.

**ZooBank LSID**. urn:lsid:zoobank.org:act:4CCDF5D8-1EFE-4CFE-AB9B-104EBDF100BF

***Rutrizoma donoghuei*** Li & Cai sp. nov. (figure 1 and electronic supplementary material, figures S1–S5)

**Material**. Holotype: NIGP166143; paratypes (5): NIGP166142, NIGP205681 (FXBA10102), NIGP205682-1, NIGP205683, NIGP205684; all mid-Cretaceous (upper Albian to lower Cenomanian), from amber mine near Noije Bum Village, Hukawng Valley, Tanai Township, Myitkyina District, Kachin State, northern Myanmar.

**Etymology**. The species is named after the evolutionary biologist Dr Philip C. J. Donoghue.

**Differential diagnosis**. *Rutrizoma donoghuei* differs from *R. pisanii* in the smaller body size (about 4.1 mm long in the holotype and similar in all the paratypes) and the edge of abdominal declivity with projected lobes at the base.

**Remarks**. The density of setae on the abdominal declivity varies across specimens. However, as the setae may fall off in life, here we decide to provisionally place all these specimens in a single species.

**ZooBank LSID**. urn:lsid:zoobank.org:act:8B0B85CB-14E9-4805-B8F0-6740C72AAC34

**Figure 2.**
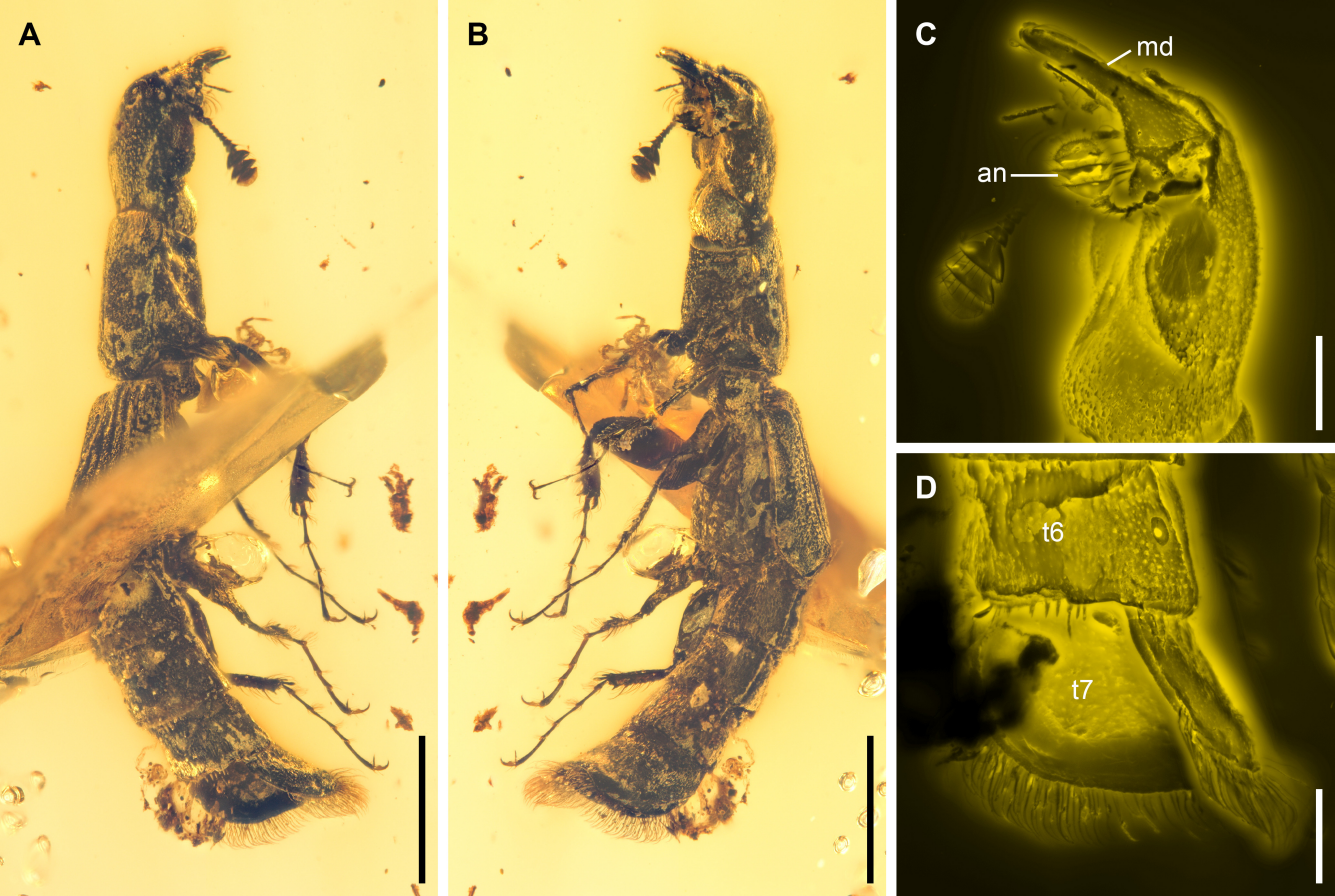
*Rutrizoma pisanii* sp. nov., NIGP205685-1, under brightfield (A,B) or confocal (C,D) microscopy. (A) Dorsolateral view. (B) Ventrolateral view. (C) Head, ventrolateral view. (D) Abdominal declivity, dorsolateral view. Abbreviations: an, antenna; md, mandible; t6−7, abdominal tergites VI–VII. Scale bars: 1 mm in (A,B); 300 μm in (C,D).

***Rutrizoma pisanii*** Li & Cai sp. nov. (figure 2 and electronic supplementary material, figure S6)

**Material**. Holotype: NIGP205685-1, mid-Cretaceous (upper Albian to lower Cenomanian), from amber mine near Noije Bum Village, Hukawng Valley, Tanai Township, Myitkyina District, Kachin State, northern Myanmar.

**Etymology**. The species is named after the evolutionary biologist Dr Davide Pisani.

**Differential diagnosis**. *Rutrizoma pisanii* differs from *R. donoghuei* in the larger body size (about 5.4 mm long in the holotype) and the simple edge of abdominal declivity.

**ZooBank LSID**. urn:lsid:zoobank.org:act:0F8BD496-97DC-42CD-A5A5-C4D52B694BC6

## 3. Discussion

### (a) The systematic placement of *Rutrizoma*

The presence of a sensorial field on the antennae (figure 1E; electronic supplementary material, figure S4I) and externally closed transverse procoxal cavities (electronic supplementary material, figure S2F) support the placement of *Rutrizoma* within the extant family Trogossitidae, commonly known as bark-gnawing beetles [37]. Additional morphological traits, such as small flat compound eyes, galeae with ciliate setae, laterally open mesocoxal cavities and robust spines on the protibiae, further suggest that *Rutrizoma* belongs to a group within Trogossitinae, roughly corresponding to the former Trogossitini (e.g. *sensu* Kolibáč [37]). The presence of ciliate setae on the galea (figure 1E; electronic supplementary material, figure S4H) is particularly noteworthy, as outside of Trogossitinae, this feature is seen only in the unusual egoliine genus *Calanthosoma* Reitter [38].

**Figure 3.**
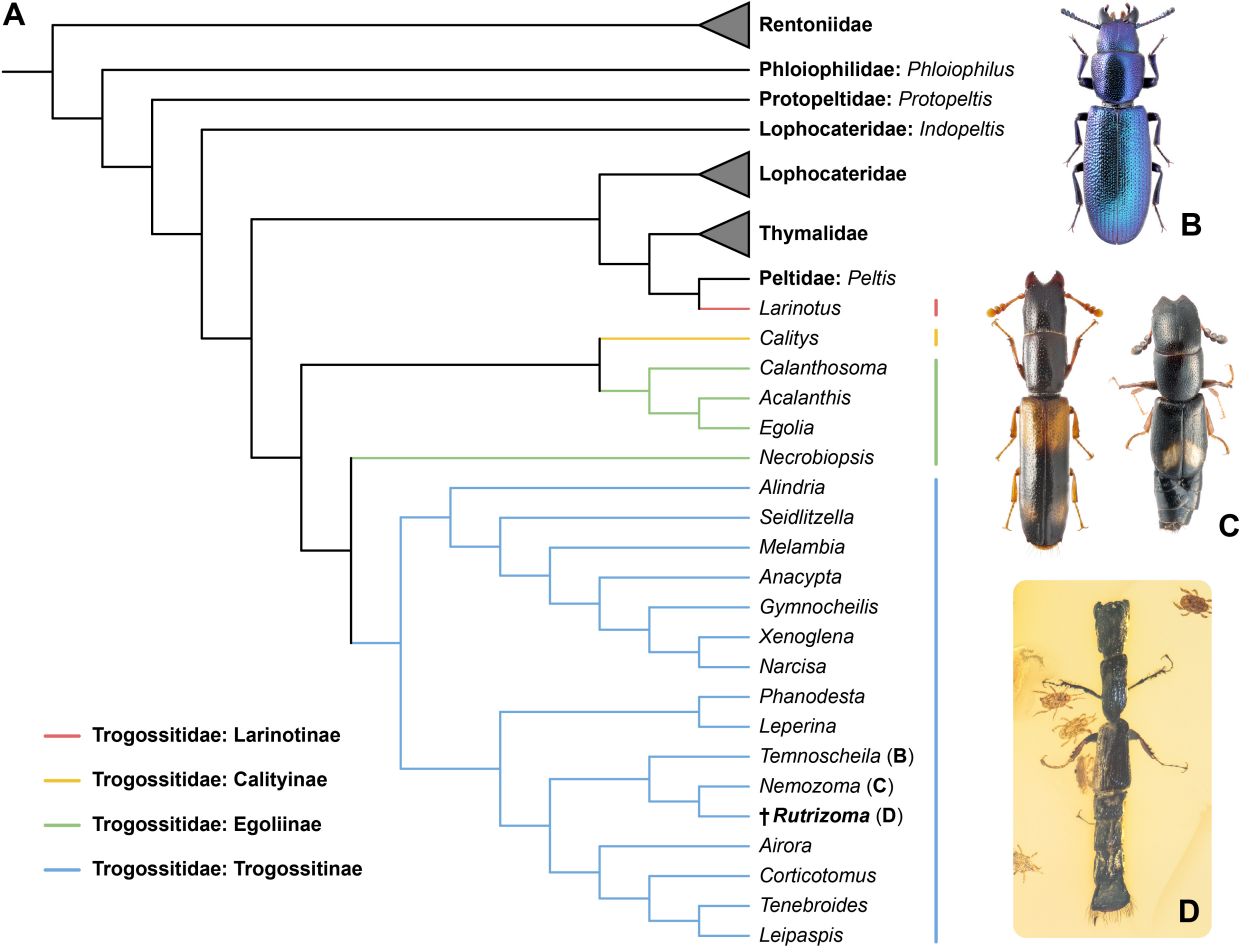
Phylogenetic placement of *Rutrizoma* and comparison with extant trogossitid genera. (A) Tree resulting from constrained parsimony analysis under implied weights. (B–D) Dorsal views of *Rutrizoma* (D) and its closest extant relatives, *Temnoscheila* (B) and *Nemozoma* (C).

*Rutrizoma* shares several key features with the extant genera *Temnoscheila* Westwood and *Nemozoma*, including a longitudinal median groove on the frons (figure 1D and electronic supplementary material, figure S7C). It also shares with *Nemozoma* the small and slender body and thin elytral epipleura [39]. Some species of *Nemozoma*, such as *N. gymnosternalis* Kolibáč and *N. schwarzi* Schaeffer, have distinctly shortened elytra, leaving part of the abdomen exposed, and also strongly convex abdominal ventrites [40–42]. The sister relationship between *Rutrizoma* and *Nemozoma* is further confirmed by our phylogenetic analysis (figure 3). However, *Rutrizoma* differs from *Nemozoma* in the absence of paired horn-like processes on the frons. Also, unlike most brachelytrous cleroids, including those of *Nemozoma*, the apical edge of elytra is straight-lined in *Rutrizoma*, much like that seen in many Staphylinidae, Histeridae or Nitidulidae. Additionally, *Rutrizoma* is unique among Trogossitidae due to its metacoxae not reaching the lateral margin of metathorax (electronic supplementary material, figure S6C) and the presence of a bordered abdominal declivity (figures 1F, 2D; electronic supplementary material, figures S2D, S4E, S5E,F).

Many groups of beetles have adults with tubulate body forms that bore or inhabit their own galleries either in the soil (e.g. some osoriine rove beetles, Staphylinidae) or, more commonly, in plant tissues [43]. The diet range of these beetles includes tissue-feeding borers, predators of the borers, and to a lesser degree, fungus feeders like lenacine monotomids, also known in the Cretaceous [44]. Among the many tubulate forms, *Rutrizoma* has a superficial resemblance to the niponiine clown beetles (Histeridae) [45,46], an unrelated group of predaceous beetles hunting within the galleries created by small wood borers, such as scolytines, bostrichids and ptinids. However, *Rutrizoma* can be easily distinguished from Niponiinae by its comparatively loose antennal club, the larger number of exposed abdominal segments, and the presence of ciliate setae on the galea. The striking resemblance between *Rutrizoma*, niponiines and other similar-looking beetles is apparently due to convergent evolution, driven by the shared ecological pressures of their gallery-inhabiting lifestyles.

### (b) A predator, but also a potential prey

Almost all extant members of the subfamily Trogossitinae are predatory [37]. Most adults hunt wood-inhabiting insects, such as bark beetles (Curculionidae: Scolytinae) and auger beetles (Bostrichidae), on branches and logs, while the larvae reside and hunt beneath the bark or within galleries. However, adults of the extant *Nemozoma* (and a few other genera, e.g. *Airora* Reitter, *Corticotomus* Sharp and *Euschaefferia* Leng), with their narrowly cylindrical bodies, are specially adapted to searching for scolytine prey within bark galleries [42,47–49]. As a putative close relative of *Nemozoma* and with a similarly slender body, *Rutrizoma* probably shared a comparable feeding habit, actively seeking xylophagous prey within galleries. Its stout, unidentate mandibles (figures 1E, 2C; electronic supplementary material, figure S7D) would have been well-suited for attacking and piercing prey, supporting the hypothesis of a predatory lifestyle [50].

**Figure 4.**
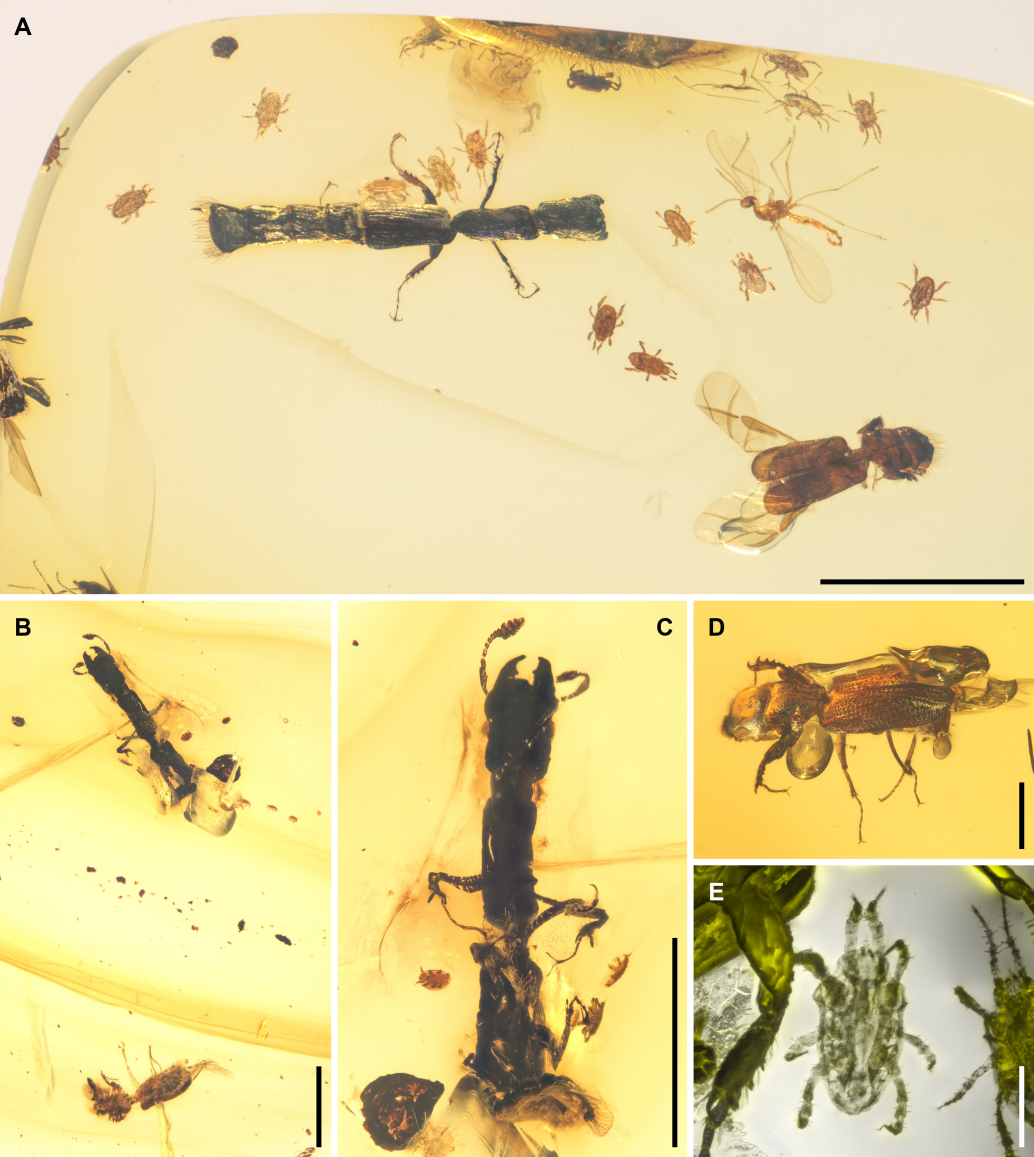
Woodboring beetles and mites associated with *Rutrizoma*, under brightfield (A–D) or confocal microscopy (E). (A) NIGP205682, syninclusion of *Rutrizoma donoghuei*, *Poinarinius aristovi* and polyaspidoid mites. (B,C) NIGP205686, syninclusion of *Rutrizoma* sp., *Poinarinius* sp., and mites. (D) NIGP205687-3, *Poinarinius aladelicatus* preserved along with two individuals of *Rutrizoma*. (E) Detail of NIGP205682, showing polyaspidoid mites associated with *Rutrizoma*. Scale bars: 2 mm in (A–C); 500 μm in (D); 200 μm in (E).

**Figure 5.**
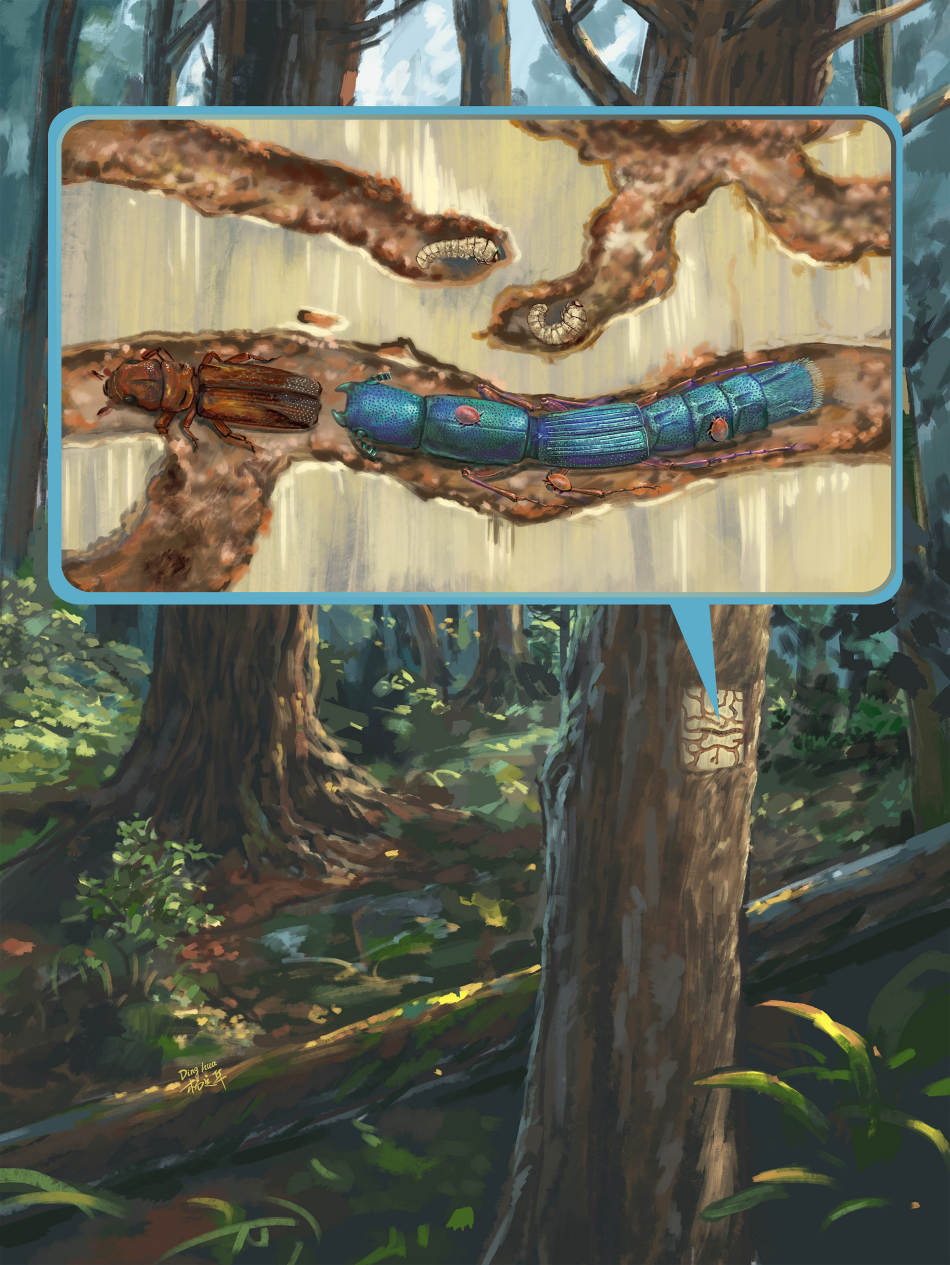
Artistic reconstruction of Cretaceous *Rutrizoma* pursuing *Poinarinius* beneath bark.

About 65% of extant beetle families have saproxylic members [51]. Wood potentially bored by beetles has been documented from Cretaceous deposits and even older strata, with evidence dating back to the Permian [52–55]. While the primary prey of *Nemozoma*, scolytines, were likely not yet widespread during the mid-Cretaceous, other woodboring beetles such as bostrichids and ptinids occupied similar ecological niches at the time [56–58]. Notably, a great diversity of *Poinarinius* Legalov (Bostrichidae) has been discovered from the same deposit (Kachin amber) that yielded *Rutrizoma* [59–61]. Many species of *Poinarinius* have morphological traits convergent with scolytines, such as a distinct or abruptly formed elytral declivity and head tubercles (figure 4D), indicating a xylophagous lifestyle in galleries. More importantly, *Rutrizoma* and *Poinarinius* have been preserved as syninclusions in four amber specimens (NIGP205682, NIGP205685, NIGP205686 and NIGP205687; figure 4A,B; electronic supplementary material, figure S8), suggesting that they likely lived in close proximity within the same forest. Based on this, we propose that *Poinarinius* may have been a potential prey for *Rutrizoma* (figure 5).

Many extant subcortical beetles, including members of Scolytinae, Platypodinae (Curculionidae), Colydiinae (Zopheridae) and Bostrichidae, as well as the Cretaceous *Poinarinius*, have a modified elytral apex with downward slope, referred to as the elytral declivity [60–66]. The elytral declivity is often decorated with sharp projections or borders. While the functions of the elytral declivities have never been seriously studied, it has been hypothesized that the elytral declivities could serve for mate recognition, effective shovelling of frass, and, particularly, defence against predators or conspecific competitors attempting to enter the gallery system [67].

Interestingly, the assumed predator of the wood-boring beetles, *Rutrizoma*, also possesses a similar bordered declivity at the abdominal, but not elytral, apex (figures 1F and 2D; electronic supplementary material, figures S2D, S4E, S5E,F). In *R. donoghuei*, the projected lobes are present at the base of the abdominal declivity. While in some modern species of *Nemozoma* the elytra may be somewhat shortened and not fully cover the abdomen [40], *Rutrizoma* displays even more specialized elytral reduction, with almost the entire abdomen exposed. This adaptation is strikingly convergent with the typical body plan of rove beetles (Staphylinidae [68]) and some cillaeine sap beetles (Nitidulidae [69]). While there may be different functions the reduction of the elytra [70], in the case of *Rutrizoma* it likely enhances flexibility and mobility within narrow galleries, though this comes at the cost of leaving the vulnerable abdomen unprotected. The abdominal declivity of *Rutrizoma* is concave and bordered by a distinct rim indicative of a gallery-inhabiting behaviour (figure 5). This feature is analogous to the phragmotic adaptations seen in wood-boring beetles like scolytines and platypodines. Phragmosis is a behavioural adaptation in which an animal employs its body as a barrier to guard its burrow. Such a phragmotic phenomenon occurs in diverse, unrelated arthropods residing in tunnels, featuring a specialized, plug-like structure adapted to obstruct the tunnel. Examples include the heads of certain ants (e.g. *Camponotus* Mayr, *Colobopsis* Mayr and *Cephalotes* Latreille), some soldier termites and multiple lineages of caddisfly larvae, and the abdominal apex of the spider *Cyclocosmia* Ausserer [71–73]. This convergently evolved structure may function as a shield, protecting *Rutrizoma* from competitors and predators. Unlike the elytral declivity found in extant wood-boring beetles, such as scolytines and bostrichids, *Rutrizoma* exhibits this feature on its abdominal apex. To our knowledge, this is the first documented instance of an abdominal phragmosis in insects (see also [74]). The distinctive declivity of *Rutrizoma* further represents the earliest unambiguous case of phragmosis in predatory beetles, exemplifying a remarkable instance of convergent evolution.

### (c) Complex ecological interactions beneath the bark of Cretaceous trees

In modern ecosystems, the galleries created by scolytine bark beetles host intricate communities where a diverse array of microbes and arthropods coexist alongside the beetles. The interactions among these organisms range from mutualisms and commensalisms to antagonism. Scolytine beetles may feed directly on the tree phloem or on the fungi thriving in their galleries [75,76]. The beetles can actively or accidentally cultivate fungi by transporting fungal spores; however, some of these fungi are inedible and may compete with other fungi that the beetles consume [77,78].

Numerous mites inhabit the galleries of beetles, hitchhiking on insects to travel across trees [79,80]. Their phoretic hosts include both scolytine beetles and their predators (electronic supplementary material, figure S9). These mites can act as either predators or parasitoids of beetle eggs and larvae [81], or as fungivores and omnivores that feed partially on fungi, occasionally assisting in fungal colonization. In some systems phoretic mites can assist their hosts by removing harmful fungal hyphae. As a result, they can directly or indirectly influence the population dynamics of scolytine beetles [77,80,82].

Many of these ecological components also existed beneath the bark of Cretaceous trees. While scolytines may not have been widespread during the mid-Cretaceous, other beetle lineages that fed at least partially on symbiotic fungi cultivated within galleries, including Lymexylidae and Bostrichidae, were already diversifying, as documented in Kachin amber [76,83].

Although fossil mites of the order Mesostigmata (Parasitiformes) have rarely been reported [84–86], the association between tortoise mites (Mesostigmata: Monogynaspida: Uropodina) and *Rutrizoma* is exceptionally well represented in our specimens. Some tortoise mites (Polyaspidoidea and Uropodoidea) disperse as deutonymphs, which are phoretic on various insects and often associated with xylophagous beetles in wood habitats [87]. Specimen NIGP205685 includes one adult of Uropodina, positioned between the first pair of legs of the *Rutrizoma* beetle (figure 3B). Specimen NIGP205682 contains 17 deutonymphs of an undescribed species near Polyaspididae (figure 4A,E), exhibiting two distinct phenotypes: a dispersal morph, characterized by a longer body and slender legs (especially legs I and IV) adapted for attachment, movement and host retention; and a sedentary morph, with a shorter body and shorter but thicker legs. Most mites in this specimen belong to the dispersal morph, with one individual attached to the *Rutrizoma* beetle. In specimen NIGP205686 (figure 4B,C), a uropodoid-like mite in dorsoventral view is a deutonymph, while a smaller nearby individual is probably a younger stage (protonymph) of the same species. Additionally, the exuviae of a larger uropodoid-like mite is present near the posterior end of the *Rutrizoma* beetle. Five other mites in lateral or frontal view may belong to a single species within Heterostigmata (Acariformes: Trombidiformes: Prostigmata: Eleutherengona). A smaller individual is likely the larval stage of this species. Although the available morphological details are insufficient for precise identification, related mites from this group have been found in Kachin amber and show evidence of symbiotic associations with fungi [88].

Our discovery suggests that a complex subcortical ecosystem had been established by the mid-Cretaceous. Moreover, subcortical environments represent an interesting context in which key specialization intersects with general stability. The subcortical realm is typically stable, at least until the host wood completely decomposes and a new tree needs to be found. Indeed, while wood-inhabiting beetles have evolved a range of complex specializations, such as the phragmotic body-form presented here or the paedogenesis of Micromalthidae [89], they also include some of the more common examples of bradytely [90]. From Cretaceous amber alone there are many examples of wood-inhabiting beetles, such as those from Jacobsoniidae [91], Monotomidae [44] and Zopheridae [92]. Living under bark affords a habitat freed from at least some of the selective pressures of the external world, but necessitates a pathway of specialization in physiology, diet and body form. Such adaptations are likely unforgiving and represent a trade-off, one that may prove fatal to a lineage when confronted with extreme changes (e.g. extreme climatic events and concomitant loss of suitable host plants, new predators, parasitoids and pathogens). Reconstructing these ancient communities and understanding how evolutionary pressures shaped them is only beginning to emerge through palaeontological research, enabled in part by the exceptional fidelity of preservation offered by amber.

## 4. Material and methods

### (a) Materials

The Kachin amber (Burmese amber) specimens studied herein originated from amber mines near Noije Bum (26°20′ N, 96°36′ E), Hukawng Valley, Kachin State, northern Myanmar. The ten specimens are deposited in the Nanjing Institute of Geology and Palaeontology (NIGP), Chinese Academy of Sciences, Nanjing, China. Individual amber pieces were trimmed with a saw mounted on a handheld rotary tool, ground with emery paper of different grit sizes, and finally polished with polishing powder.

### (b) Fossil imaging

Brightfield images were taken with a Zeiss Discovery V20 stereo microscope. Confocal images were obtained with a Zeiss LSM710 confocal laser scanning microscope, using the 561 nm (DPSS 561-10) laser excitation line [93]. Images were stacked with Helicon Focus 7.0.2, Zerene Stacker 1.04 and Adobe Photoshop CC, and were further processed in Adobe Photoshop CC to adjust brightness and contrast. Microtomographic data for the specimens NIGP166143, NIGP205681, NIGP205685-1 and NIGP205688 were obtained with a Zeiss Xradia 520 Versa 3D X-ray microscope at the micro-CT laboratory of NIGP and analysed in VGStudio MAX 3.0.

While high-resolution imaging provides precise morphological data, we also include an artistic reconstruction of the Cretaceous subcortical community to aid in visualizing ecological interactions and to support broader science communication.

### (c) Phylogenetic analysis

We conducted a constrained morphology-based phylogenetic analysis under maximum parsimony to evaluate the systematic placement of the new fossil genus. The data matrix for extant genera was taken from Li *et al.* [94], which was derived from Kolibáč [38,50]. The full matrix includes 61 adult and 32 larval characters, among which we successfully coded 28 adult characters for the new fossil. The constraining backbone tree was the same as the one used by Li *et al.* [95], which was created based on the Bayesian molecular tree under the site-heterogeneous CAT-GTR model by Li *et al.* [94]. For taxa with both morphological and molecular data, their interrelationships were fixed as the backbone tree. The fossil genus and other extant taxa without molecular data were allowed to move freely across the backbone tree. The analysis was performed under implied weights in R 4.1.0 [96], using the R script provided by Li *et al.* [95], which deploys the R package TreeSearch 1.3.1 [97]. The concavity constant was set to 12, following the suggestion by Goloboff *et al.* [98] and Smith [99]. The resulting tree (figure 3; electronic supplementray material, figure S10) was visualized with the online tool iTOL 6.6 [100] and graphically edited with Adobe Illustrator CC 2017.

## Data Availability

The data for the phylogenetic analysis and supplementary figures are available on Figshare [101]. The original confocal and micro-CT data are available on Zenodo [102]. Supplementary material is available online [103].
